# Distribution of blood lead and cadmium levels in healthy children aged 0 to 18 years and analysis of related influencing factors in Henan, China: data findings from 2017 to 2022

**DOI:** 10.1186/s13052-024-01614-z

**Published:** 2024-03-07

**Authors:** Hui Yan, Bo Zhai, Ruiling Feng, Penggao Wang, Fang Yang, Yang Zhou

**Affiliations:** 1 Henan Provincial Clinical Research Center for Pediatric Diseases, Henan Key Laboratory of Pediatric Genetics and Metabolic Diseases, Children’s Hospital Affiliated to Zhengzhou University, Henan Children’s Hospital, Zhengzhou Children’s Hospital, Zhengzhou, 450018 China; 2 Department of Cardiothoracic Surgery, Children’s Hospital Affiliated to Zhengzhou University, Henan Children’s Hospital, Zhengzhou Children’s Hospital, Zhengzhou, 450018 China

**Keywords:** Lead, Cadmium, Blood lead levels, Blood cadmium levels, Influencing factors, Children

## Abstract

**Background:**

There is still a lack of data on blood lead levels (BLLs) and blood lead levels (BLLs) in healthy children of all ages from 0 to 18 years in China. This study was performed to analyze the BLLs and BCLs in healthy children aged 0-18 years from 2017 to 2022 in urban and rural areas of Henan Province, Central China, as well as their relationships with socio-demographic variables and certain relevant exposure factors. To provide a basis for evaluating public health policy development and exposure risk management.

**Methods:**

This was an observational study containing data from 17 prefecture-level cities in Henan, China. Blood Pb and Cd levels were determined using a triple quadrupole inductively coupled plasma mass spectrometer equipped with an autosampler. We first calculated the concentrations of Pb and Cd elements in participants of different genders, ages and years, and then created visual graphs depicting the distribution of each element in terms of gender, age and year (2017-2022). The rates between different groups were compared using the Chi-square test or Fisher exact test (if applicable). The means were compared by one-way ANOVA, medians were compared with the Kruskal-Wallis rank-sum test. Generalized linear models (GLM) were performed to estimate the effects of various factors on blood Pb and Cd concentrations in children.

**Results:**

We recruited a total of 25,920 children (16,142 boys and 9,778 girls) aged 0.01 to 18.00 years (2.58 (1.00,6.25)). The median of BLLs was 23.48µg/L, around 9.39% of studied children had elevated BLLs. The median of BCLs was 0.66µg/L, around 1.84% of studied children had elevated BCLs. The median blood Pb concentration was higher in boys (23.90µg/L) than in girls (22.75µg/L) (*P*<0.001). The median blood Pb concentration was highest in the 3-7 years group (24.51µg/L) and the median blood Cd concentration was highest in the 1-3 years group (0.66µg/L) among all age groups. Both BLLs and BCLs were substantially higher in children in 2020-2022 compared to 2017-2019. Rural children had lower BLLs and higher BCLs. The results of the generalized linear model showed that children in households using Oil, coal, pellet or other wood as a fuel for heating, children with higher frequency of exposure to tobacco smoke and beverage intake had significantly increased chances of elevated BLLs and BCLs.

**Conclusions:**

Pb and Cd exposure of children in this area is relatively low, but associated risk factors continue to exist in vulnerable populations. This study is the first big data analysis of Pb and Cd in children in Henan, China, and provides baseline information for future research.

## Introduction

With the development of modern industry and transportation, heavy metal pollutants are gradually exposed and released, and heavy metal pollution in the environment is becoming increasingly serious, and the resulting harm to human health has caused widespread concern. Heavy metals in the environment can enter the human body through a variety of pathways, and children in particular are a susceptible population to environmental pollution [1]. Many studies have revealed that children are more sensitive to heavy metal toxicity because they are in the early stages of rapid growth and development, and their blood-brain barrier and immune system are not yet sufficiently developed [2–4]. Thus, long-term heavy metal exposure will have adverse effects on children's intelligence and growth and development, which will easily lead to multiple damages in children [2–4].

Heavy metals lead (Pb) and cadmium (Cd) are toxic and harmful substances widely present in production and living environment, causing environmental pollution and also seriously threatening human health. Lead and cadmium are the two heavy metals that pose the greatest threat to children's health in the environment [4]. The International Agency for Research on Cancer (IARC) has classified lead and cadmium as human carcinogens (Group I), and childhood Pb poisoning and Cd poisoning have become a global public health problem that has caused widespread concern worldwide [4–6]. The effects of Pb exposure on children's health are multifaceted and include intellectual impairment [7], cognitive deficits [8], adverse behavioral outcomes [9], low birth weight/height [10], and negative consequences on bone composition and development [3], which can be observed even at low levels of exposure [11]. In 2012, the United State (US) Centers for Disease Control and Prevention (CDC) Advisory Committee on Childhood Lead Poisoning Prevention concluded that there was no safe level of Pb exposure and revised the reference value from 100µg/L to 50µg/L. This reference value was based on 97.5% of blood lead concentrations in the US National Health and Nutrition Examination Survey (NHANES) population and served as a trigger point to guide clinical and public health interventions [12]. Afterwards, 35µg/L was recommended to the CDC as a cut-off value based on NHANES survey data from 2011-2014, and this concentration is still under review [13]. Cadmium is a toxic heavy metal that has no physiological role in the human body and can cause damage to many systems such as bone, nerve, immune, respiratory, circulatory, urinary and reproductive. Studies have indicated that Cd intake can increase the risk of cancer and death and lead to kidney damage and decrease bone mineral density, which can lead to diseases such as osteochondrosis and osteoporosis [14]. Low blood cadmium levels (BCLs) may be associated with adverse neurodevelopmental outcomes or neurobehavioral manifestations [15]. There is also no available reference safe level of Cd exposure in children.

In China, with the phasing out of leaded gasoline in 2000, blood lead levels (BLLs) in children dropped dramatically, such a conclusion was made by reviewing blood Pb (PbB) studies conducted across China [16]. However, some relevant sources of Pb exposures in children still persist, such as exposure through food, drinking water, tobacco smoke and contaminated dust particles [17]. Cadmium is readily absorbed by various plants, such as vegetables, grains and tobacco. Tobacco smoke is volatile and can be effectively absorbed after inhalation and is a major source of exposure [17]. From a public health perspective, there is an urgent need for adequate information on adverse events related to Pb and Cd exposure. Blood samples are still one of the most commonly accepted options for laboratory analysis to assess recent and past exposure to chemical contaminants. Whole blood has been the preferred biofluid for assessing Pb exposure, both for screening and diagnosis and for long-term biomonitoring. BLL remains the most reliable indicator of recent Pb exposure [9]. Cd enters the body and binds mainly to blood cells, and BCL can represent recent Cd exposure [18].

Most of the available studies on BLLs and BCLs in children have focused on areas with Pb and Cd contamination [19, 20]. Alternatively, studies have been conducted on non-healthy children (usually a group of children with certain diseases) [21], or have monitored the results of the survey only for a certain age group [16–18]. There is still a lack of data on BLLs and BCLs in healthy children of all ages from 0 to 18 years in China. This study was performed to analyze the BLLs and BCLs in healthy children aged 0-18 years from 2017 to 2022 in urban and rural areas of Henan Province, Central China, as well as their relationships with socio-demographic variables and certain relevant exposure factors. To provide a basis for evaluating public health policy development and exposure risk management.

## Materials and methods

### Study design and population

This cross-sectional study was conducted from January 1, 2017, to December 30, 2022, at the Medical Examination Center of Children's Hospital Affiliated to Zhengzhou University, Henan Province, China. It is a tertiary children's health care institution integrating medical treatment, health care, public health, teaching, research and training, and the largest specialized children's hospital in Henan Province. The hospital is located in Zhengzhou, the capital of Henan Province, which is an important central city in central China. The children and guardians participating in the study were from 17 prefecture-level cities in Henan Province. The children who presented to the hospital underwent routine health examinations, while a standardized questionnaire [22] was employed to collect relevant sociodemographic factors, including: sociodemographic variables, children's daily habits, home heating methods, and annual household income. The questionnaire was administered by the pediatrician during the patient's visit, while the child's blood was collected.

The study subjects inclusion criteria were: i) good health status as assessed by two pediatricians at the medical examination center, ii) no trace elements or mineral supplements within six months, iii) complete laboratory examinations and questionnaire data, and iv) consent of the children themselves or their guardians to participate in the study. Subjects with CRP levels at or above 0.29 mg/dL were excluded in order to prevent an influence on the results due to inflammation. We recruited a total of 25,920 children (16,142 boys and 9,778 girls) aged 0.01 to 18.00 years (mean=3.78, SD=3.25). The study was reviewed and approved by the Ethics Committee of Zhengzhou University, and all respondents signed informed consent. The whole process and all methods of the study were performed in accordance with the relevant guidelines and regulations for the construction of clinical diagnosis and treatment and clinical research ethics review committees.

### Collection and determination of blood samples

Blood samples were collected at the Medical Examination Center of Children's Hospital Affiliated to Zhengzhou University and transported to the ISO Class 6 clean room of the key pediatric laboratory for analysis. After each participant fasted for more than 8 hours, venous blood samples were taken by a trained nurse using a lithium heparin vacuum collection tube designed for trace element assays, followed by gentle mixing. The mouth of the tube was sealed tightly to prevent leakage and contamination at the time of delivery. If the blood sample could not be submitted in time, the blood sample was refrigerated and stored at 2-8℃ and the test was completed within 72h.

A 100 µL blood was drawn from each sample using a micropipette, which was melted, vortexed, and diluted in a 1:25 (v/v) solution of 0.5% (v/v) HNO_3_ and 0.05% (v/v) Triton X-100 (Sigma, USA). Blood Pb and Cd levels were determined using a triple quadrupole inductively coupled plasma mass spectrometer (Q-ICPMS, PerkinElmer, NexION® 350D, USA) equipped with an autosampler (PerkinElmer, model AS-93 plus, USA). Daily analytical conditions were optimized to meet the requirements of the analysis.

For data quality control, every 20 samples were run once using certified reference materials (Seronorm™ Trace Elements Whole Blood Level-2 and Level-3, Norway) with prepared blood to assess the accuracy and precision of the method. The limits of detection (LOD) and quantifcation (LOQ) of the method were calculated as 3- and 10-fold the standard deviation of the concentrations of 11 independent blank replicates multiplied by the corresponding dilution multiple. In this study, the LOD of the method was 0.14 µg/L for Pb and 0.04 µg/L for Cd, and the LOQ of the method was 0.62 µg/L for Pb and 0.06 µg/L for Cd, respectively. The intra- and inter-day precisions were 2.0% and 5.8% for Pb, and 1.1% and 4.9% for Cd. The reference intoxication levels in this laboratory were as follows: Pb > 50µg/L and Cd > 2.5µg/L.

### Statistical analysis

The continuous variables were statistically described by mean ($\overline{X }$) and standard deviation (*SD*) and median (*M*) and quartile (Q_1_~Q_3_); categorical variables were described by composition ratio and rate. The participants were divided into five groups according to their ages: Group A: 0~1 (age <1 year, *N* = 5745); Group B: 1~3 (1 ≤ age < 3 years, *N* = 8049); Group C: 3~7 (3 ≤ age < 7 years, *N* = 6811); Group D: 7~13 (7 ≤ age < 13 years, *N* = 4998); Group E: 13~18 (13 ≤ age ≤ 18 years, *N* = 317). We first calculated the concentrations of Pb and Cd elements in participants of different genders, ages and years, and then created visual graphs depicting the distribution of each element in terms of gender, age and year (2017-2022) (presented as box plots). In addition, we evaluated how many children had an overconcentration of Pb and Cd compared to the reference concentrations. The rates between different groups were compared using the Chi-square test or Fisher exact test (if applicable). The means were compared by one-way ANOVA; medians were compared with the Kruskal-Wallis rank-sum test. Generalized linear models (GLM) were performed to estimate the effects of various factors on blood Pb and Cd concentrations in children. All statistical analyses and drawing process were performed using software SAS.9.4 and SPSS 27.0. *P* values < 0.05 (two-sided) were considered statistically significant, unless indicated otherwise.

## Results

A total of 25,920 study subjects meeting the inclusion and exclusion criteria were included in this study, of which 62.3% were boys, and the median age was 2.58 (1.00,6.25) years. After grouping by age, the largest proportion of children in the 1 to 3 years group and the smallest proportion of children in the 13 to 18 years group were among all study participants. A total of 5843 children were enrolled in 2017, 5099 in 2018, 4421 in 2019, 3659 in 2020, 3765 in 2021, and 3133 in 2022. The differences in the distribution of gender, age, ethnicity, frequent playgrounds, heating fuel, frequency of staying in rooms with smokers, frequency of non-alcoholic beverage intake, and annual household income were statistically significant between years (*P*<0.001), and there were no differences in the distribution of the study population's place of residence. The details of the sociodemographic characteristics of the sample of children in this study were presented in Table 1.

**Table 1:** Demographics of the children who participated in the study

	**Total** (*N*=25920)	**Year**							
		**2017** (*n*=5843)	**2018** (*n*=5099)	**2019** (*n*=4421)	**2020** (*n*=3659)	**2021** (*n*=3765)	**2022** (*n*=3133)	*H/X*^*2*^	*P*
**Gender**(n,(%))									
Boys	16142(62.3)	3667(62.8)	3200(62.8)	2810(63.6)	2306(63.0)	2316(61.5)	1843(58.8)	21.864	**<0.001**
Girls	9778(37.7)	2176(37.2)	1899(37.2)	1611(36.4)	1353(37.0)	1449(38.5)	1290(41.2)		
**Age (years)**(*M*(*Q*_*1*_,*Q*_*3*_))(n,(%))	2.58 (1.00,6.25)	2.25 (0.88,5.67)	2.75 (1.08,6.58)	3.08 (1.17,6.92)	2.42 (1.00,5.83)	2.42 (1.08,6.17)	2.83 (1.08,5.92)	180.997	**<0.001**
A[0~1)	5745(22.2)	1634(28.0)	1049(20.6)	837(18.9)	902(24.7)	710(18.9)	613(19.6)	296.243	**<0.001**
B[1~3)	8049(31.1)	1690(28.9)	1582(31.0)	1279(28.9)	1131(30.9)	1371(36.4)	996(31.8)		
C[3~7)	6811(26.3)	1470(25.2)	1320(25.9)	1230(27.8)	957(26.2)	931(24.7)	903(28.8)		
D[7~13)	4998(19.3)	982(16.8)	1070(21.0)	1015(23.0)	630(17.2)	709(18.8)	592(18.9)		
E[13~18]	317(1.2)	67(1.1)	78(1.5)	60(1.4)	39(1.1)	44(1.2)	29(0.9)		
**Ethnicity**(n,(%))									
Han nationality	24504(94.5)	5450(93.3)	4723(92.6)	4200(95.0)	3514(96.0)	3603(95.7)	3014(96.2)	98.516	**<0.001**
Other Minorities	1416(5.5)	393(6.7)	376(7.4)	221(5.0)	145(4.0)	162(4.3)	119(3.8)		
**Region of residence**(n,(%))									
Urban area	17413(67.2)	3880(66.4)	3426(67.2)	2948(66.7)	2480(67.8)	2564(68.1)	2115(67.5)	4.287	0.509
Rural area	8507(32.8)	1963(33.6)	1673(32.8)	1473(33.3)	1179(32.2)	1201(31.9)	1018(32.5)		
**Where the child plays**(n,(%))									
At home	11221(43.3)	1922(32.9)	1637(32.1)	1473(33.3)	2115(57.8)	2229(59.2)	1845(58.9)	708.972	**<0.001**
Outdoors	14699(56.7)	3921(67.1)	3462(67.9)	2948(66.7)	1544(42.2)	1536(40.8)	1288(41.1)		
**Fuel for heating**(n,(%))									
Oil, coal, pellet or other wood	7040(27.2)	1525(26.1)	1315(25.8)	1176(26.6)	1006(27.5)	1088(28.9)	930(29.7)	24.905	**<0.001**
Other fuels	18880(72.8)	4318(73.9)	3784(74.2)	3245(73.4)	2653(72.5)	2677(71.1)	2203(70.3)		
**Staying in rooms at home where people smoke**(n,(%))									
Never	10309(39.8)	2583(44.2)	2136(41.9)	1880(42.5)	1464(40.0)	1300(34.5)	946(30.2)	59.792	**<0.001**
Not daily	7975(30.8)	1794(30.7)	1606(31.5)	1347(30.5)	1145(31.3)	1143(30.4)	940(30.0)		
Daily	7636(29.4)	1466(25.1)	1357(26.6)	1194(27.0)	1050(28.7)	1322(35.1)	1247(39.8)		
**Non-alcoholic beverage intake**(n,(%))									
Never	6475(25.0)	1730(29.6)	1453(28.5)	1053(23.8)	838(22.9)	790(21.0)	611(19.5)	32.051	**<0.001**
Not daily	16083(62.0)	3523(60.3)	2998(58.8)	2785(63.0)	2331(63.7)	2410(64.0)	2036(65.0)		
Daily	3362(13.0)	590(10.1)	648(12.7)	583(13.2)	490(13.4)	565(15.0)	486(15.5)		
**Annual household income**(n,(%))									
≤ 60K	8220(31.7)	1753(30.0)	1448(28.4)	1233(27.9)	1288(35.2)	1370(36.4)	1128(36.0)	48.808	**<0.001**
> 60K	17700(68.3)	4090(70.0)	3651(71.6)	3188(72.1)	2371(64.8)	2395(63.6)	2005(64.0)		

**Fig. 1:**
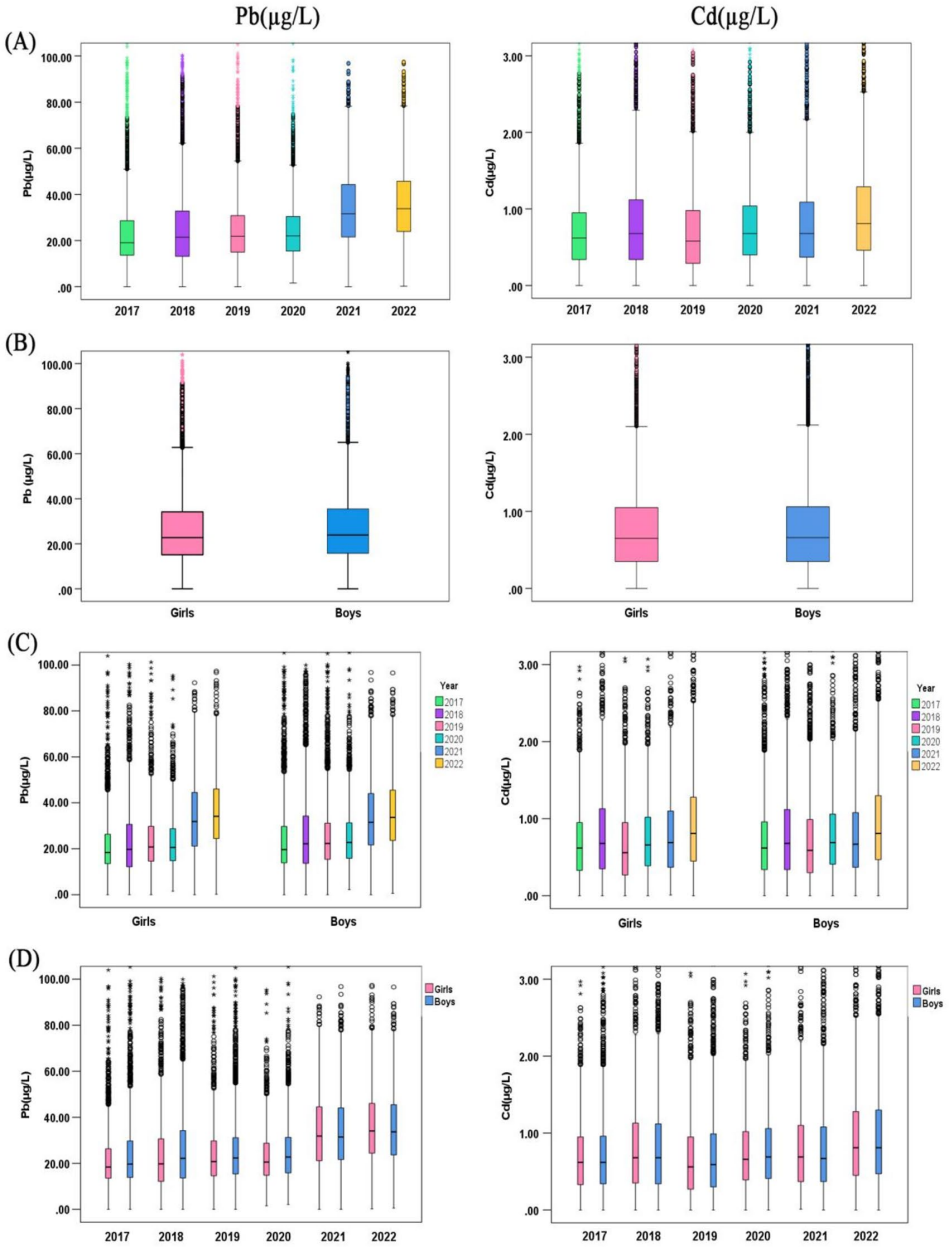
Blood levels of elemental lead(Pb) and cadmium(Cd) in the children aged 0~18 years by gender, 2017-2022. **A** Results of aggregated data by year. **B** Results of aggregated data by gender. **C** Results by year for different genders. **D** Results by gender in different years. The boxplot showed the median values of elements and their percentile ranges

**Fig. 2:**
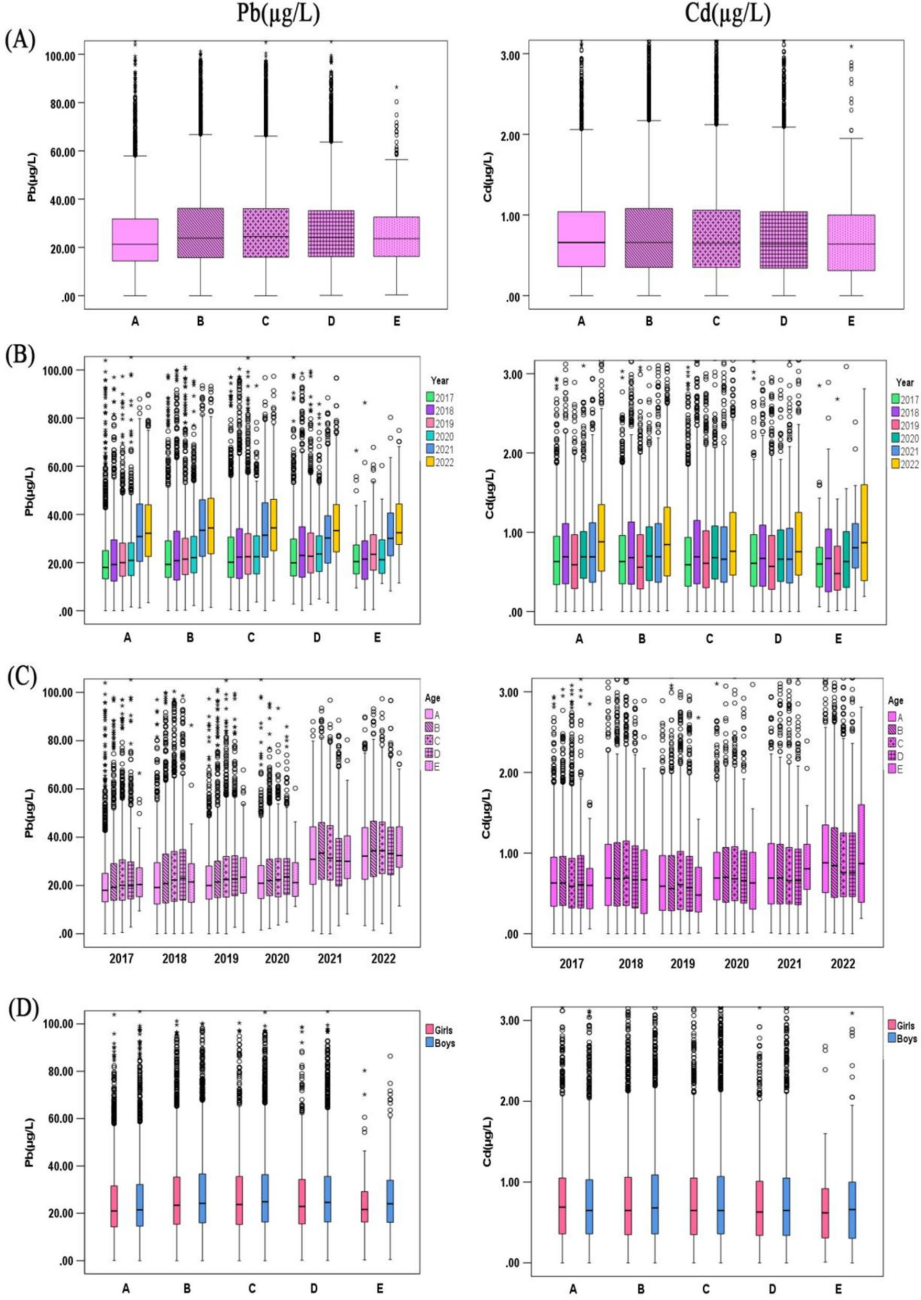
Blood levels of elemental lead(Pb) and cadmium(Cd) in the children aged 0~18 years by age, 2017-2022. **A** Results of aggregated data by age. **B** Results by year for different ages. **C** Results by age in different years. **D** Results by gender in different age groups. The boxplot showed the median values of elements and their percentile ranges. A: 0~1 years group, B: 1~3 years group, C: 3~7 years group, D: 7~13 years group, E: 13~18 years group

Table 2 revealed that the overall median blood Pb concentration of the included study subjects was 23.48µg/L and the median blood Cd concentration was 0.66µg/L. The median blood Pb concentration was higher in boys (23.90µg/L) than in girls (22.75µg/L) (*P*<0.001). The difference between the median blood Cd concentrations of boys and girls was not statistically significant (*P*=0.489). Figure 1B visualized the distribution of blood Pb and blood Cd concentrations by gender. We also analyzed the distribution of blood Pb and Cd concentrations by gender under different years (Fig. 1D), and by gender in different age subgroups (Fig. 2D). It was also observed that the blood Pb concentration was higher in boys than in girls regardless of the grouping. In addition, the median blood Pb concentration and median blood Cd concentration varied with age group and year. Figures 1A and 2A graphically demonstrated the distribution of blood Pb and blood Cd concentrations by year and age groups, and the differences were statistically significant. From 2017 to 2022 roughly, the median blood Pb concentrations showed an increasing trend, with the greatest rising in 2021 and reaching the highest (33.80µg/L) by 2022. And this increase in blood Pb concentrations over the years was present in both the boy and girl groups when stratified by gender (Fig. 1C). The increase in blood Pb concentrations over time was also observed in all age groups when stratified by age group (Fig. 2B). In contrast, median blood Cd concentrations was lowest in 2019 (0.58µg/L) and then increased to reach a maximum in 2022 (0.81µg/L). This was also the case for both the boy and girl groups after stratification by gender (Fig. 1C). This variation was also present in all age groups after stratification by age group (Fig. 2B). The median blood Pb concentrations was highest in the 3-7 years group (24.51µg/L) and the median blood Cd concentrations was highest in the 1-3 years group (0.66µg/L) among all age groups. When stratified by year, the observations differed, with the highest median blood Pb concentrations in the 1 to 3 years group and the highest median blood Cd concentrations in the 13 to 18 years group in both 2021 and 2022 (Fig. 2C).

**Table 2:** Comparison of elemental lead(Pb) and cadmium(Cd) levels in the blood of children age from 0 to 18 in Henan, China, based on gender, age, and year

**Group**	**N**	**Pb(µg/L)**	*H*	*P*	**Cd(µg/L)**	*H*	*P*
**Gender**							
Boys	16142	23.90(15.81,35.50) 28.23±19.63	5.635	**<0.001**	0.66(0.35,1.06) 0.79±0.60	0.693	0.489
Girls	9778	22.75(15.11,34.20) 27.17±19.29			0.65(0.35,1.05) 0.79±0.59		
**Age (years)**							
A[0~1)	5745	21.27(14.39,31.82)^a,b,c,d^ 25.90±20.28	150.006	**<0.001**	0.66(0.36,1.04)^c,d^ 0.78±0.57	11.055	**0.026**
B[1~3)	8049	23.85(15.74,36.16)^a^ 28.92±21.99			0.66(0.35,1.08)^g,h^ 0.80±0.60		
C[3~7)	6811	24.51(15.94,36.04)^b^ 28.28±17.56			0.65(0.35,1.06) 0.80±0.61		
D[7~13)	4998	24.19(16.19,35.23)^c^ 27.75±16.78			0.64(0.34,1.04)^c,g^ 0.78±0.62		
E[13~18]	317	23.63(16.19,32.61)^d^ 26.42±14.59			0.64(0.31,1.00)^d,h^ 0.74±0.61		
**Year**							
A(2017)	5843	19.09(13.69,28.62)^a,b,c,d,e^ 24.10±18.98	689.523	**<0.001**	0.62(0.34,0.95)^a,b,c,d,e^ 0.71±0.52	416.896	**<0.001**
B(2018)	5099	21.45(13.16,32.80)^a,h,i^ 26.11±22.60			0.68(0.34,1.12)^a,f,i^ 0.82±0.66		
C(2019)	4421	21.90(15.01,30.84)^b,k,l^ 25.56±17.71			0.58(0.29,0.98)^b,f,j,k,l^ 0.72±0.58		
D(2020)	3659	22.06(15.45,30.41)^c,m,n^ 24.82±13.96			0.68(0.40,1.04)^c,j,n^ 0.79±0.54		
E(2021)	3765	31.62(21.53,44.31)^d,h,k,m,o^ 34.41±18.66			0.68(0.37,1.09)^d,k,o^ 0.80±0.58		
F(2022)	3133	33.80(23.92,45.72)^e,i,l,n,o^ 36.37±19.57			0.81(0.46,1.29)^e,i,l,n,o^ 0.96±0.70		
Total	25920	23.48(15.53,34.98) 27.83±19.51			0.66(0.35,1.05) 0.79±0.60		

Results were expressed as median (P_25,_ P_75_); Kruskal-Wallis was used to compare differences in two and more groups. The mean values and SDs were noted in the second row for each element

^a^A difference between the group A and B, *P*<0.05

^b^A difference between the group A and C, *P*<0.05

^c^A difference between the group A and D, *P*<0.05

^d^A difference between the group A and E, *P*<0.05

^e^A difference between the group A and F, *P*<0.05

^f^A difference between the group B and C, *P*<0.05

^g^A difference between the group B and D, *P*<0.05

^h^A difference between the group B and E, *P*<0.05

^i^A difference between the group B and F, *P*<0.05

^j^A difference between the group C and D, *P*<0.05

^k^A difference between the group C and E, *P*<0.05

^l^A difference between the group C and F, *P*<0.05

^m^A difference between the group D and E, *P*<0.05

^n^A difference between the group D and F, *P*<0.05

^o^A difference between the group E and F, *P*<0.05

Next, we calculated the overconcentration rate of the elements Pb and Cd in difffferent gender, age and year groups. Taking Pb > 100µg/L as the standard reference for Pb poisoning, 0.50% of boys and 0.47% of girls had higher concentrations of Pb (Table 3). In addition, blood Pb over 50µg/L could be considered excessive Pb for children due to the critical period of childhood, and 9.73% of boys and 8.85% of girls met these criteria; the difference was significant. When blood Cd concentrations > 2.5µg/L were used as the threshold, 1.9% of boys and 1.75 of girls were above this concentration and the difference was not statistically significant. Between different age groups, the children in Group B (1~3 years group) had a relatively higher rate of Pb > 150µg/L, Pb > 100µg/L and Pb > 50µg/L, and the difference in rates between the age groups was statistically significant. And the children in Group E (13~18 years group) had a relatively higher rate of Cd > 2.5µg/L, but the difference in rates between the age groups was not statistically significant. When 50µg/L was taken as the reference value for BLLs, the lowest rate of exceedance (6.58%) was found in children in 2019 and the highest rate (18.16%) in 2022, the difference in rates between the year groups was statistically significant. When 2.5µg/L was taken as the reference value for BCLs, the lowest rate of exceedance (0.98%) was found in children in 2017 as well as 2020 and the highest rate (3.99%) in 2022, the difference in rates between the year groups was statistically significant.

**Table 3:** The rates of elevated elemental lead(Pb) and cadmium(Cd) in different groups (n(%))

	**N**	**Pb>150 (µg/L)**	**Pb>100 (µg/L)**	**Pb>50 (µg/L)**	**Cd>5 (µg/L)**	**Cd>2.5 (µg/L)**
**Gender**						
Boys	16142	41(0.25)	81(0.50)	1570(9.73)	0(0.00)	307(1.90)
Girls	9778	21(0.21)	46(0.47)	865(8.85)*	1(0.01)	171(1.75)
**Age (years)**						
A[0~1)	5745	14(0.24)^#^	25(0.44)^#^	435(7.57)^#^	0(0.00)	89(1.55)
B[1~3)	8049	32(0.40)^#^	69(0.86)^#^	854(10.61)^#^	0(0.00)	136(1.69)
C[3~7)	6811	10(0.15)^#^	21(0.31)^#^	700(10.28)^#^	0(0.00)	144(2.11)
D[7~13)	4998	6(0.12)^#^	12(0.24)^#^	424(8.48)^#^	1(0.02)	101(2.02)
E[13~18]	317	0(0.00)^#^	0(0.00)^#^	22(6.94)^#^	0(0.00)	8(2.52)
**Year**						
A(2017)	5843	19(0.33)^&^	42(0.72)^&^	327(5.60)^&^	0(0.00)	57(0.98)^&^
B(2018)	5099	20(0.39)^&^	36(0.71)^&^	434(8.51)^&^	0(0.00)	129(2.53)^&^
C(2019)	4421	4(0.09)^&^	19(0.43)^&^	291(6.58)^&^	0(0.00)	70(1.58)^&^
D(2020)	3659	3(0.08)^&^	9(0.25)^&^	179(4.89)^&^	1(0.03)	36(0.98)^&^
E(2021)	3765	9(0.24)^&^	12(0.32)^&^	635(16.87)^&^	0(0.00)	61(1.62)^&^
F(2022)	3133	7(0.22)^&^	9(0.29)^&^	569(18.16)^&^	0(0.00)	125(3.99)^&^
**Total**	25920	62(0.24)	127(0.49)	2435(9.39)	1(0.00)	478(1.84)

Results were expressed as number of cases and percentage (%). Chi-square test or Fisher’s exact test was used to compare differences between groups. *P < 0.05, compared with boys. ^#^The difference in rates between the age groups was statistically significant, *P* < 0.05. ^&^The differences in rates between years were statistically significant, *P* < 0.05

Finally, we analyzed the correlates that may affect BLLs and BCLs in children employing GLM (Table 4). The results indicated that gender, age, place of residence, heating fuel, frequency of staying in rooms with smokers, and frequency of non-alcoholic beverage intake had an effect on blood Pb concentration in children. Some factors that had influenced blood Cd concentration in children included place of residence, heating fuel, frequency of staying in rooms with smokers, and frequency of non-alcoholic beverage intake. Girls had lower BLLs (*OR*: 0.52, 95%*CI*: 0.24-0.89). BLLs were at greatest risk of elevation in the 1~3 years old group (*OR*: 1.92, 95%*CI*: 1.23-2.85) , but decreased after 13 years (*OR*: 0.93, 95%*CI*: 0.87-0.99) in children. Compared to urban areas, children in rural areas showed lower BLLs (*OR*: 0.85, 95%*CI*: 0.68-0.96) but higher BCLs (*OR*: 1.19, 95%*CI*: 1.03-1.58). The consumption of oil, coal, pellet or other wood as heating fuel in the home was a risk factor for elevated BLLs (*OR*: 1.76, 95%*CI*: 1.14-2.88) and BCLs (*OR*: 2.05, 95%*CI*: 1.34-3.71) in children. Children who spent daily in rooms with smokers were more likely to have elevated BLLs (*OR*: 2.37, 95%*CI*: 1.41-3.92) and BCLs (*OR*: 2.59, 95%*CI*: 1.48-4.06) than children who never spent time in rooms with smokers. Higher BLLs (*OR*: 2.12, 95%*CI*: 1.27-3.84) and higher BCLs (*OR*: 2.18, 95%*CI*: 1.29-3.87) were also more likely to be observed in those who consumed non-alcoholic beverages on a daily basis. Meanwhile, we also presented the above results more visually with graphs.

**Table 4:**
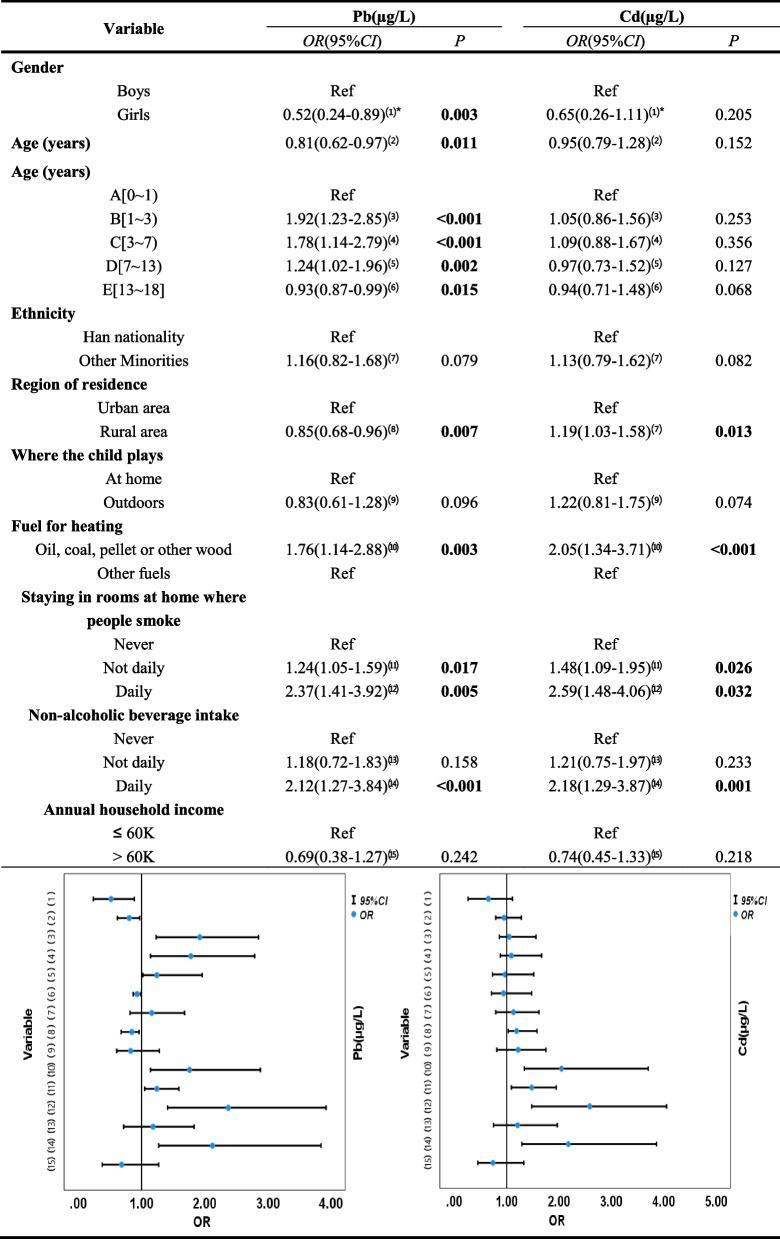
Generalized linear model analysis of related factors affecting blood lead and cadmium concentrations in children.

^*^The superscript number of the variable corresponds one to one with the variable number in the confidence interval graph. *OR* Odds ratio, *CI* Confidence interval

## Discussion

There are currently no reference ranges for blood lead and blood cadmium for healthy children in China. Mostly, lead poisoning was empirically determined by blood Pb > 100µg/L and cadmium poisoning by blood Cd > 5µg/L. In addition, differences in biological substrates, age ranges, assays, and geographic regions in published studies made comparisons difficult. In our study, the median BLL of children was 23.48µg/L, ranging from 19.09µg/L to 33.80µg/L between 2017 and 2022. It was lower than the data from researches in Italy (34.00µg/L) [23], São Paulo, Brazil (31.20µg/L) [18], and Iran (24.90µg/L) [24], and lower than the reported data from Nanjing (41.16µg/L) [25], Jinan (49.42µg/L) [26], Beijing (42.8µg/L) [27], Nanning (52.6µg/L) [28], Changchun (60.29µg/L) [29], Wuhan (33.72µg/L) [30] and Hunan (35.00µg/L) [31]. However, it was higher than the data reported for children in the United States (12.00µg/L) [32], children in Spain (11.00µg/L) [22] and adolescents in Sweden (7.10µg/L) [33]. Overall, a total of 0.49% of children suffered from BLL > 100µg/L, which was lower than Nanjing (1.3%) [25], Jinan (1.4%) [26], Changchun (10%) [29] and higher than Hunan (0.33%) [31]. Meanwhile, the median BCL of children was 0.66µg/L, ranging from 0.62µg/L to 0.81µg/L between 2017 and 2022. Our result was lower than the data reported in Jinan (1.47µg/L) [26], Changchun (1.26µg/L) [29] and Hunan (1.00µg/L) [30], China. But it was higher than the results reported by Liu et al. (0.65µg/L or 0.39µg/L) [34], as well as those from Korea (0.30µg/L) [35], Brazil (0.48µg/L) [18] and Germany (0.06µg/L) [36]. The above results indicated that with the introduction of the policy of banning the use of leaded gasoline in 2000, the local government had attached great importance to the publicity and prevention of children's lead exposure, such as the restriction of lead paint and industrial emissions, and also reflected people's attention to healthy living environment and awareness. Nonetheless, our reported data still fell short of the desired level when compared to developed countries such as Europe and the United States. The public health issue of Pb and Cd exposure remains of great concern, given that Pb and Cd exposures originate from a variety of routes, mainly from contaminated air, dust, food, water or jewelry, inhalation of cigarette smoke, or improper handling of the metals themselves [34].

In the present study, both BLLs and prevalence of Pb poisoning (BLL > 50µg/L) were higher in boys compared to girls, similar to other reports [28, 30, 31]. This may be due to higher erythrocyte pressure levels in boys with more sources of Pb exposure [34]. In children, dust is a significant source of Pb exposure, whether it is house dust or ground dust that reach the mouth due to habits such as sucking hands or even licking the surface of an object or placing an object in the mouth [37]. BLLs and BCLs were usually higher in toddlers and school-age children than in infancy and older children due to the natural hand-to-mouth contact activities of toddlers and school-age children and the resulting increased exposure to Pb and Cd through soil and dust [22], which was consistent with the data of our study. The BLLs and BCLs of children in this study varied between years. The year 2020 appeared to be a turning point, after which both BLLs and BCLs in children were more substantially elevated. It was possible that the non-pharmacological interventions measures including restrictions on travel and outdoor activities [38] for severe acute respiratory syndrome coronavirus 2 (SARS-CoV-2), which was first detected in 2020, kept children indoors with their families most of the time. Children had an increased probability of being exposed to materials used for interior decorating, fuels used for cooking and heating, tobacco smoke from family members' smoking, and ingesting snacks such as beverages. More rigorous conclusions need to be substantiated by more researches.

In this study, we found that rural children had lower BLLs and higher BCLs. It might be due to the higher traffic density, more serious air pollution, easier deposition of outdoor dust, more fancy home decoration materials, and more varied children's toys in urban areas, resulting in the higher probability of children's exposure to lead-contaminated environments. Despite the ban on adding Pb to gasoline, Pb did not break down, so the millions of tons of Pb released into the air from the use of leaded gasoline had contaminated the soil and remained in the air at very low concentrations, especially in urban areas [22]. Besides tobacco, food was the main source of Cd exposure in children [14]. Rural children might pay less attention to food health and hygiene than urban children. And some chemical factories were usually built in suburban areas near rural areas, discharging cadmium-containing effluent, resulting in Cd contamination of soil, which might lead to high Cd content in local crops in the long run. Our study showed that children in households using Oil, coal, pellet or other wood as fuel for heating had higher BLLs and BCLs. This was probably attributed to the fact that the gases and particulate matter emitted from the combustion of these fuels increased the suspension time and concentration of Pb and Cd in the air [39]. Previous studies have reported high levels of arsenic, cadmium, chromium, nickel and lead in Chinese cigarettes [40]. Our results also indicated that children exposed more frequently to tobacco smoke had higher BLLs and BCLs. Many studies have revealed a correlation between BLLs and smoking, with a strong correspondence between Pb levels in air and Pb levels in tobacco, and a decrease with decreasing Pb levels in gasoline [22, 41]. In addition, it has been reported that tobacco plants are readily enriched for Cd and that tobacco smoke exposure affects Cd and Pb much more than other metals [42]. Intestinal absorption of Pb and Cd is susceptible in children than in adults, and Cd is excreted slowly through the kidneys [22]. Our results showed that children who ingested beverages more frequently had higher BLLs and BCLs. Beverage intake might affect micronutrient balance in the body, while deficiencies in calcium, iron, zinc and protein increased intestinal absorption of Pb and Cd [43]. Moreover, beverages contained a large number of food additives, coloring and preservatives, these substances into the human body were easy to aggravate the burden on the kidneys to affect the excretion of Pb and Cd.

There were several limitations to this study. Firstly, this was a cross-sectional design and we were unable to establish a causal relationship between risk factors and BLLs and BCLs. Secondly, the fact that the study sample consisted of the pediatric population of the medical examination center clinic limited its external validity, and it was worth considering whether the data obtained could be extrapolated to the general population. Finally, information on the variables collected was limited and some confounders affecting outcomes might not have been included. The advantages of this study were the high analytical sensitivity of the method for the determination of Pb and Cd in children's blood, with reference to international quality standards, which ensured the accuracy of the results. Data management staff were highly qualified, data collection work was organized and planned, and the data collected were relatively accurate. The study population included children of all ages from 0 to 18 years old. The large sample size of this study reduced selection bias, and the results can provide scientific basis for further intervention by local governments and hospitals.

## Conclusion

In this study, we assessed lead and cadmium levels in the blood of 25,920 children aged 0-18 years from 2017 to 2022 in Henan, China. The median of BLLs was 23.48µg/L, around 9.39% of studied children had elevated BLLs. The median of BCLs was 0.66µg/L, around 1.84% of studied children had elevated BCLs. Pb and Cd exposure in this area was relatively low in this study. Boys had higher BLLs and prevalence of Pb poisoning (BLL > 50µg/L) compared to girls. BLLs and BCLs were usually higher in toddlers and school-age children than in infancy and older children. Both BLLs and BCLs were substantially elevated in children in 2020-2022 compared to 2017-2019. Rural children had lower BLLs and higher BCLs. Children in households using Oil, coal, pellet or other wood as a fuel for heating, with higher frequency of exposure to tobacco smoke and beverage intake had significantly increased chances of elevated BLLs and BCLs. These results can provide more attention and corresponding strategies for parents of high-risk children. To the best of our knowledge, this is the first big data analysis of Pb and Cd elements in healthy children in Henan, China. Awareness of children's BLLs and BCLs and their exposure risks provides health policymakers with the information necessary to develop public health measures, community-based preventive interventions, and to promote children's health. It also provides baseline data for future researches.

## Data Availability

The datasets used and/or analysed during the current study are available from the corresponding author on reasonable request.

## Abbreviations

Pb: Lead
Cd: Cadmium
IARC: International Agency for Research on Cancer
US: United State
CDC: Centers for Disease Control and Prevention
NHANES: National Health and Nutrition Examination Survey
BCLs: Blood cadmium levels
BLLs: Blood lead levels
PbB: Blood Pb
LOD: Limits of detection
LOQ: Limits of quantifcation
GLM: Generalized linear models
OR: Odds ratio
CI: Confidence interval
SARS-CoV-2: Severe acute respiratory syndrome coronavirus 2
